# Towards precision oncology in angiosarcomas using next generation “omic” technologies

**DOI:** 10.18632/oncotarget.27996

**Published:** 2021-09-14

**Authors:** Grace Fangmin Tan, Jason Yongsheng Chan

**Affiliations:** ^1^Division of Medical Oncology, National Cancer Centre Singapore, Singapore; ^2^Oncology Academic Clinical Program, Duke-NUS Medical School, Singapore

**Keywords:** rare cancer, single cell sequencing, chemoresistance, spatial transcriptomics

## Abstract

Angiosarcomas are a group of aggressive tumors of vascular origin. Although thought to be a rare cancer constituting just 1–2% of all soft tissue sarcomas, recent observations suggest that angiosarcomas are more common amongst Asian populations as compared to the West, suggesting the possibility of distinct genetic or environmental triggers influencing its pathogenesis. Advances in genomic sequencing efforts have led to the discovery of ultraviolet mutation signatures and high tumor mutation burden as common features of angiosarcoma of the head and neck. In addition, multi-omic analyses integrated with clinical data identified 3 subtypes characterized by distinctive etiological and biological phenotypes, with potential implications on precision therapy. The systemic and local immune milieu, as well as the presence of “giant” tumor cells, was also recently demonstrated to influence clinical behavior and patient outcomes, further highlighting complexities of this disease. Improvements in next generation “omic”-based technologies are expected to improve our understanding of angiosarcoma and guide the development of precision oncology in this rare cancer.

## INTRODUCTION

Angiosarcomas are a group of aggressive mesenchymal tumors of endothelial origin. Although rare, the clinical features and predisposing factors have been fairly well-described in the literature. Notably, the biological heterogeneity of this disease entity is manifested through its diverse forms of clinical presentation and behavior. At the anatomical level, angiosarcoma most often presents as a cutaneous malignancy in the head and neck region, but may also originate from other sites including the breast, viscera, trunk and extremity. In terms of etiology and risk factors, angiosarcoma arises either as primary *de novo* cases, or secondary to radiation exposure or other medical conditions [1]. Curiously, it has recently been observed that angiosarcomas are more common amongst Asian populations as compared to the West, suggesting the possibility of distinct genetic or environmental triggers influencing its pathogenesis. Until recently, little progress has been made in the understanding of its molecular and genetic composition, and their relevance to clinical application is only beginning to emerge.

Using next generation sequencing, The Angiosarcoma Project - a large patient-partnered US-Canadian research initiative, presented evidence for ultraviolet mutagenesis as a common feature of angiosarcomas of the head and neck. This characteristic molecular signature underlies high tumor mutation burden (TMB), a potential biomarker for deriving benefit from checkpoint immunotherapy [2]. This was also similarly observed in an Asian cohort of angiosarcoma patients, albeit in only half the cases. Interestingly, further to identifying ultraviolet mutational signatures, the examination of oncogenic signaling pathways by gene expression profiling and the immune microenvironment using multiplex immunohistochemistry allowed the discovery of three unique cluster subtypes characterized by distinctive etiological and biological phenotypes [3]. Cluster 1 was composed of predominantly primary angiosarcomas with relatively lower oncogenic and inflammatory signals. Cluster 2 was characterized by an upregulation of epigenetic and oncogenic signaling pathway genes, and consisted the majority of secondary angiosarcomas. Cluster 3 was enriched in inflammation-related pathways and immune cells, and harbored high Tumor Inflammation Signature (TIS) scores, in contrast to the immune “cold” microenvironment of cluster 1 tumors. Signatures of ultraviolet mutagenesis and high TMB, as well as tumors arising from the head and neck region, were predominantly found in clusters 1 and 3. The clinical implications of these observations are immediately evident, since both high TMB and high TIS scores have been reported to confer superior response rates to checkpoint immunotherapy [4]. In parallel, targeted agents against epigenetic targets such as BET or DNMT inhibitors, or those inhibiting oncogenic kinases, may show selective activity in cluster 2 cases.

The prognosis of patients with angiosarcoma remains dismal, and patients with advanced disease usually do not survive beyond a year despite treatment with conventional chemotherapy or targeted agents. Determinants of survival typically include traditional clinical parameters such as age, tumor size, and stage of disease [5], while predictors of chemotherapy response are essentially unknown. In a previous study on 712 patients diagnosed with various soft tissue sarcomas, we had shown that an elevated circulating peripheral blood neutrophil-to-lymphocyte ratio (NLR) was associated with aggressive biological features and poor clinical outcomes [6]. Expanding on these observations, we demonstrated in a recent study that high NLR was predictive of worse overall survival in patients with angiosarcoma, alongside older age and presence of distant metastasis. Using these 3 factors (“MAN” score), patients could be risk-stratified into low (0–1), intermediate (2), and high (3) risk subgroups based on the number of risk features [7].

Conventional cytotoxic chemotherapy with anthracycline or taxane-based regimens are currently the most widely used in angiosarcomas, and while treatment response has been shown to improve survival, predictive biomarkers to guide treatment decisions are lacking. By examining the intra-tumoral immune responses and oncogenic signals, it was shown that the dysregulation of several immune-oncology pathways, including angiogenesis, matrix remodeling and metastasis, cytokine and chemokine signaling, as well as an accumulation of neutrophils and macrophages, resulted in primary chemoresistance [7].

Apart from tumor microenvironment, tumor heterogeneity is also thought to contribute to primary chemoresistance and relapse. We examined resected angiosarcoma specimens and angiosarcoma cell-lines for giant cells, a rare (< 1%), bizarre subpopulation of malignant cells found across various tumor types and thought to contribute to treatment resistance [8]. Giant cells were found in 41.4% of primary angiosarcoma specimens, as well as angiosarcoma cell lines MOLAS and ISOHAS. In patients, we found that the presence of giant cells conferred poorer overall survival as well as poorer response to cytotoxic chemotherapy, while giant cells demonstrated relative *in vitro* chemoresistance in comparison to regular angiosarcoma cells. The detailed analysis of single cell populations of giant cells and regular angiosarcoma cells revealed similar genomic landscapes, reaffirming their status as tumor-derived rather than infiltrating immune or stromal cells. Interestingly, when examined in the context of intra-tumoral immune signals, the presence of giant cells also correlated with immune pathway dysregulation including matrix remodeling and metastasis, cytokine and chemokine signaling, hinting at a complex interplay between tumor heterogeneity, dysregulated tumor microenvironment and chemoresistance which warrants further investigation.

How do we progress research in a rare cancer like angiosarcoma from here? Emerging next generation “omic” technologies beyond bulk whole genomic or transcriptomic sequencing are expected to dissect the composition of the tumor microenvironment of angiosarcoma at higher resolution [9]. Single cell sequencing coupled with spatial profiling technologies will facilitate our understanding of the complex interplay between tumor cells, tumor infiltrating immune cells, and the stromal components. Such high definition data, correlated with clinical information, will further enable the discovery of novel biomarkers required for the achievement of precision oncology in angiosarcoma.
